# Effects of Dietary Lactoferrin–Osteopontin Complexes on Immune Function, Intestinal Health and Microbiota Composition in Kittens

**DOI:** 10.3390/vetsci13080805

**Published:** 2026-08-14

**Authors:** Min Liu, Zeming Song, Shenghong Zhang, Hehe Liu, Fushi Li, Renxia Yin, Xiaoe Xiang, Lian Li

**Affiliations:** 1 College of Animal Science and Technology, Nanjing Agricultural University, No. 1 Weigang, Nanjing 210095, China; 2024805118@stu.njau.edu.cn (M.L.); 2025805126@stu.njau.edu.cn (H.L.); 2025805127@stu.njau.edu.cn (F.L.); 2025105017@stu.njau.edu.cn (R.Y.);; 2 Hangzhou Keyun Biotechnology Research Institute Co., Ltd., No. 259 Pinglan Road, Hangzhou 311200, China

**Keywords:** lactoferrin–osteopontin complex, kittens, immunity, antioxidant capacity, gut microbiota

## Abstract

This study evaluated the effects of dietary supplementation with a commercial lactoferrin–osteopontin complexes preparation (LF-OPN; LF:OPN mass ratio 8:1; 240 mg/kg body weight) on growth, immune indices, antioxidant status, intestinal barrier-related markers, fecal microbiota, short-chain fatty acids (SCFAs), peripheral lymphocyte subsets, and the whole-blood transcriptome of growing British Shorthair kittens. Sixteen kittens were randomly assigned to either a basal diet group (CON) or a group supplemented with LF-OPN complexes (LFT) for 28 days. LF-OPN supplementation did not significantly affect body weight or average daily gain. On D28, the LFT group had a higher serum immunoglobulin M concentration and a lower tumor necrosis factor-α concentration (*p* < 0.05). Serum superoxide dismutase and glutathione peroxidase activities and total antioxidant capacity were higher, whereas malondialdehyde was lower (*p* < 0.05) in the LFT group (*p* < 0.01). Fecal 16S rRNA gene sequencing revealed no significant difference in alpha diversity; howerer, LEfSe identified selective taxonomic difference, including higher relative abundances of Holdemanella and Erysipelotrichaceae and lower relative abundances of Enterobacteriaceae and Escherichia-Shigella in the LFT group. Fecal short-chain fatty acid concentrations were unchanged. Flow cytometry revealed a higher proportions of peripheral CD4+ lymphocytes and a higher CD4+/CD8+ ratio in the LFT group (*p* < 0.01), whereas the CD8+ proportion was unchanged. Whole-blood RNA sequencing identified 283 differentially expressed genes (199 upregulated and 84 downregulated), with enrichment of immune-related pathways including hematopoietic cell lineage, antigen processing and presentation, cytokine–cytokine receptor interaction, NF-κB signaling, and IL-17 signaling, but these findings were not independently validated. The tested LF-OPN preparation was well tolerated and was associated with elevated antioxidant status, altered immune indices, and selective modulation of the fecal microbiota in growing kittens. The study does not establish synergy or identify the individual contributions of either protein.

## Simple Summary

Young kittens have developing gastrointestinal and immune systems, which may increase their susceptibility to environmental stress and infection. Lactoferrin (LF) and osteopontin (OPN) are bioactive milk proteins with complementary antimicrobial and immunoregulatory activities and roles in intestinal-development functions; they, and that can associate to form an LF-OPN complex. In this study, 16 British Shorthair kittens received either a basal diet or the same diet supplemented for 28 days with a commercial LF-OPN preparation (LF:OPN mass ratio, 8:1; 240 mg/kg body weight). Supplementation did not adversely affect growth. Kittens receiving LF-OPN had higher serum immunoglobulin M, lower tumor necrosis factor-α, improved antioxidant indices, a higher proportion of circulating CD4+ lymphocytes, and a higher CD4+/CD8+ ratio. The preparation also selectively altered fecal bacterial taxa, decreasing Enterobacteriaceae and Escherichia-Shigella while enriching several taxa identified by LEfSe, although overall microbial diversity and fecal short-chain fatty acid concentrations remained unchanged. Whole-blood transcriptomics analysis identified changes in immune-related pathways, but these exploratory findings need independent verification. These observed changes support further evaluation of the LF-OPN complexes as a promising functional ingredient in kitten nutrition. Because LF-only, OPN-only, and uncomplexed LF + OPN groups were not included, the contributions of the individual proteins and any synergistic effect cannot be determined.

## 1. Introduction

The kitten stage, particularly between 3 and 4 months of age, is characterized by rapid somatic growth, immune maturation, and establishment of the gut microbial ecosystem [1,2,3]. During this period, maternally derived antibody concentrations decline while endogenous immune competence and intestinal barrier function continue to mature, potentially increasing susceptibility to environmental changes, infection, and nutritional stress [1,4,5]. The gut microbiota contributes to nutrient metabolism, epithelial barrier maintenance, and mucosal immune regulation and responds to dietary composition and format [6,7,8,9,10]. Studies in growing kittens have shown that the dietary protein-to-carbohydrate ratio and diet format alter fecal microbial composition and metabolic output [4,11,12]. These observations support the use of targeted nutritional strategies during early life, although controlled studies in kittens remain limited.

Lactoferrin (LF) is a multifunctional iron-binding glycoprotein naturally present in mammalian milk that exhibits antibacterial, antiviral, immunomodulatory, and anti-inflammatory properties [13,14,15]. Osteopontin (OPN) is a highly phosphorylated, integrin-binding glycoprotein involved in early development, immune regulation, intestinal maturation, and tissue remodeling [16,17,18]. LF and OPN coexist in the milk matrix and can associate as non-covalent heteroprotein complexes under physiological ionic conditions [19]. The bovine LF-OPN complex has also been shown to promote intestinal epithelial proliferation through PI3K/Akt signaling [20], and recent studies using intestinal organoids and ex vivo fermentation models suggest that bovine LF-OPN complex formats can influence intestinal uptake and microbial metabolism [21]. Bioactivity has also been demonstrated in infant-formula and other in vitro models [22,23].

Dietary LF has been reported to modulate immune indices, intestinal morphology, microbial communities, and antioxidant status in piglets, dogs, mice and rats [24,25,26,27]. OPN-enriched formula has also altered circulating lymphocyte subsets and cytokine responses in infants [28,29]. However, evidence regarding dietary LF-OPN complexes in companion animals is scarce, and their effects in growing kittens have not been reported. Therefore, this study evaluated a commercial LF-OPN preparation (LF:OPN mass ratio, 8:1; 240 mg/kg body weight) for its effects on growth, humoral and cellular immune indices, antioxidant status, intestinal barrier-related markers, fecal microbiota and SCFAs, and the peripheral whole-blood transcriptome of young British Shorthair kittens. The preparation was evaluated as a complete commercial product; the study was not designed to distinguish the individual effects of LF and OPN, characterize the degree of complexation, or test for synergy.

## 2. Materials and Methods

### 2.1. Animals and Experimental Design

The animal experiment was approved by the Animal Welfare and Ethics Committee of Nanjing Agricultural University (Approval No.: NJAU.No20250119012). All British Shorthair kittens underwent clinical examinations and parasite testing by a licensed veterinarian before enrollment.

Sixteen healthy British Shorthair kittens, aged 3 to 4 months, were assigned to two groups using a randomized grouping process designed to minimize differences between groups: the basal diet group (CON group; *n* = 8; 4 males and 4 females) and the group fed basal diet supplemented with LF-OPN complexes at 240 mg/kg BW (LFT; *n* = 8; 4 males and 4 females). The nutritional composition of the diet is presented in Table 1. The dose of 240 mg/kg body weight was selected based on the manufacturer’s instructions. The dose was used as an exploratory supplementation level rather than an established optimal dose. LF-OPN (vetwish^®^) is a commercially available ingredient containing lactoferrin and osteopontin at a mass ratio of 8:1. The powdered supplement was evenly distributed over the diet surface immediately before feeding. The entire experiment lasted for 35 days, comprising a 7-day adaptation period followed by a 28-day experimental period. Body weights and fecal scores were recorded weekly. All animals were housed in compliance with applicable animal welfare regulations. The housing environment was maintained under controlled conditions with an ambient temperature of 22–26 °C, a relative humidity of 50–65%, and a 12 h light/dark cycle. Enclosures were cleaned and disinfected daily, and environmental stress was minimized through consistent handling schedules and the provision of environmental enrichment.

### 2.2. Sample Collection

Blood samples were collected from the cephalic vein on D0 and D28. For hematological analysis, 0.1 mL of blood was transferred into EDTA-coated vacuum tubes. Additional 1.0 mL blood samples were centrifuged at 3500 rpm for 15 min at 4 °C; the resulting serum was transferred into sterile Eppendorf tubes and stored at −20 °C until further analysis.

On D28, a 1.0 mL blood sample was collected from each kitten for peripheral blood mononuclear cell (PBMC) isolation and serum separation; the PBMCs were subsequently used for flow cytometric analysis. Simultaneously, a 0.5 mL blood sample was collected and transferred into a PAXgene^®^ Blood RNA Tube (762165, BD Biosciences, Franklin Lakes, NJ, USA) for peripheral whole-blood transcriptomic analysis. Approximately 5 g of freshly voided feces was aseptically collected from each kitten into sterile tubes. Samples were stored at −80 °C until 16S rRNA gene sequencing and SCFA analysis.

### 2.3. Measurement of Serum Parameters

Serum immunoglobulin A (IgA), immunoglobulin M (IgM), immunoglobulin G (IgG), interleukin-1β (IL-1β), interleukin-6 (IL-6), tumor necrosis factor-α (TNF-α), lipopolysaccharide (LPS), diamine oxidase (DAO), and C-reactive protein (CRP) were measured using feline-specific ELISA kits. Superoxide dismutase (SOD), glutathione peroxidase (GSH-Px), total antioxidant capacity (T-AOC), and malondialdehyde (MDA) were measured using biochemical assay kits. All assays were performed according to the manufacturers’ protocols (Nanjing Heyuan Biotechnology Co., Ltd., Nanjing, China).

### 2.4. Fecal Microbiota Analysis

Total bacterial DNA was extracted from kitten fecal samples, and DNA integrity was assessed using 1% agarose gel electrophoresis. DNA extraction and sequencing were conducted by Shanghai Ling-En Biotechnology Co., Ltd. (Shanghai, China). The V3-V4 hypervariable regions of the 16S rRNA gene were amplified using primers 341F (5’-CCTACGGGNGGCWGCAG-3’) and 806R (5’-GACTACHVGGGTATCTAATCC-3’). The PCR amplicons were excised from 2% agarose gels and purified using the AxyPrep DNA Gel Extraction Kit (Axygen Biosciences, Union City, CA, USA) according to the manufacturer’s instructions. The purified products were quantified using the QuantiFluor™-ST Blue Fluorescence Quantitative System (Promega Corporation, Madison, WI, USA). Equimolar amounts of purified amplicons were pooled and subjected to paired-end sequencing (2 × 250 bp) on an Illumina platform. For microbial community analysis, alpha diversity indices (observed species, ACE, Chao1, Shannon, and Simpson), were calculated to assess species richness and evenness. Beta diversity was estimated based on Bray–Curtis distances and visualized by principal coordinate analysis (PCoA), and overall community structure was compared between groups using PERMANOVA based on Bray–Curtis distances. Linear discriminant analysis effect size (LEfSe) was used to identify microbial taxa that differed significantly in abundance between treatment groups (LDA score > 2, *p* < 0.05).

### 2.5. Determination of Fecal Short-Chain Fatty Acids (SCFAs)

Fecal SCFA concentrations were determined using gas chromatography–mass spectrometry (GC-MS) at Shanghai Ling-En Biotechnology Co., Ltd. (Shanghai, China). Working standards were prepared in methanol using individual SCFA standards. A 100 mg fecal sample was weighed and mixed with 100 µL of 15% phosphoric acid. Then 100 µL of internal standard solution (isocaproic acid) and 400 µL of diethyl ether were added. The mixture was vortexed for 1 min and centrifuged at 12,000 rpm for 10 min at 4 °C. The supernatant was analyzed using an Agilent 6890N/5975B GC-MS system (Agilent Technologies, Santa Clara, CA, USA).

### 2.6. Flow Cytometric Analysis of PBMC Subsets

Fresh whole blood was collected into EDTA-coated vacuum tubes and diluted 1:1 with phosphate-buffered saline (PBS; SH30256.01, HyClone, Logan, UT, USA) at room temperature. The diluted blood was carefully layered onto Ficoll-Paque PREMIUM (17144002, Cytiva, Marlborough, MA, USA) in a 15 mL conical tube and centrifuged at 400× *g* for 20 min at room temperature (20 °C), with acceleration and deceleration set to 1. The PBMC layer was collected and washed twice with PBS at 300× *g* for 10 min, with acceleration and deceleration set to 9. PBMCs were resuspended at 1 × 10^7^ cells/mL in cryopreservation medium (90% FBS and 10% DMSO), aliquoted into cryovials, stored at −80 °C overnight, and then transferred to liquid nitrogen until analysis.

For flow cytometry, cryopreserved cells were rapidly thawed in a 37 °C water bath. The cell suspension was then transferred into a pre-warmed RPMI 1640 medium (11879020, Gibco, Shanghai, China) supplemented with 10% FBS and 1% penicillin–streptomycin (100 U/mL penicillin and 100 μg/mL streptomycin; Solarbio, Beijing, China). The cells were centrifuged at 300× *g* for 10 min at room temperature. The PBMCs were resuspended in RPMI 1640 at 1 × 10^7^ cells/mL and incubated with 5% BSA in PBS at 4 °C for 30 min to block nonspecific binding.

All antibodies were used according to the manufacturers’ instructions. The cells were stained with an unconjugated mouse anti-feline CD8 primary antibody (MCA1347GA, Bio-Rad, Hercules, CA, USA) at 4 °C for 60 min. After washing, the cells were incubated with an APC-conjugated goat anti-mouse IgG secondary antibody (SA00014-10, Proteintech, Wuhan, China) at 4 °C for an additional 60 min. Following another wash, the cells were blocked with PBS containing 10% mouse serum (BL1053A, Biosharp, Beijing, China) at 4 °C for 30 min, and then incubated with an FITC-conjugated mouse anti-feline CD4 antibody (MCA1346F, Bio-Rad, Hercules, CA, USA) at 4 °C for 30 min. The cells were washed twice with 500 µL of flow cytometry buffer (97% PBS and 3% FBS) at 400× *g* for 4 min. The cell pellets were resuspended in 500 µL of flow cytometry buffer, and 5 µL of propidium iodide (PI; C1008M, Beyotime, Shanghai, China) was added 10 min before analysis to exclude dead cells. Samples were acquired using a BD FACSCalibur™ flow cytometer (BD Biosciences, San Jose, CA, USA). Data were analyzed using FlowJo software (version 10.10.0).

### 2.7. RNA Extraction and Transcriptomic Sequencing

PAXgene blood samples were held at room temperature for 6–8 h before further processing. Total RNA was extracted TRIzol Reagent (Life Technologies, Carlsbad, CA, USA) according to the manufacturer’s instructions by Beijing Biomarker Technologies Co., Ltd. (Beijing, China). RNA concentration and purity were measured using a NanoDrop 2000 (Thermo Fisher Scientific, Wilmington, DE, USA), and RNA integrity was assessed using the RNA Nano 6000 Assay Kit on an Agilent Bioanalyzer 2100 system (Agilent Technologies, Santa Clara, CA, USA). A total of 1 μg RNA per sample was used as input material for library preparations. Sequencing libraries were generated using the Hieff NGS Ultima Dual-mode mRNA Library Prep Kit for Illumina (Yeasen Biotechnology [Shanghai] Co., Ltd., Shanghai, China) according to the manufacturer’s recommendations, and index codes were added to assign sequences to individual samples. Briefly, mRNA was purified from total RNA using poly-T oligo-attached magnetic beads. First-strand cDNA was synthesized, followed by second-strand cDNA synthesis. The remaining overhangs were converted into blunt ends though exonuclease/polymerase activity. After adenylation of the 3’ ends of the DNA fragments, NEBNext adaptors with hairpin-loop structures were ligated to prepare the fragments for hybridization. Library fragments were purified using the AMPure XP system (Beckman Coulter, Beverly, MA, USA). Next, 3 μL of USER Enzyme (NEB, Ipswich, MA, USA) was added to the size-selected, adaptor-ligated cDNA and incubated at 37 °C for 15 min, followed by 5 min at 95 °C before PCR. PCR amplification was then performed using Phusion High-Fidelity DNA polymerase, Universal PCR primers, and Index (X) Primer. Finally, PCR products were purified using the (AMPure XP system) and library quality was assessed using the Agilent Bioanalyzer 2100 system. The libraries were sequenced on an Illumina NovaSeq platform to generate 150 bp paired-end reads, according to the manufacturer’s instructions.

Differential expression analysis between groups was performed using the DESeq2 R package (version 1.30.1). DESeq2 provides statistical routines for analyzing differential expression in digital gene expression data using a negative binomial distribution model. The resulting *p* values were adjusted using the Benjamini–Hochberg method to control the false discovery rate. Genes with an *p* < 0.05 and |log_2_FC| > 1.5 found by DESeq2 were considered differentially expressed. KEGG pathway enrichment analysis of the DEGs was performed using the ClusterProfiler R package (version 4.4.4). Because the RNA-seq findings were not independently validated by RT-qPCR, all transcriptomic analyses were interpreted as exploratory.

### 2.8. Statistical Analysis

Data were analyzed using IBM SPSS Statistics software (version 27.0; IBM Corp., Armonk, NY, USA). Differences in SCFA concentrations and lymphocyte subsets between groups were analyzed using independent samples t-test. Repeated-measures ANOVA was used to assess differences in the other variables between the two groups, with Bonferroni correction applied for multiple comparisons. Data are expressed as the mean ± standard error of the mean (SEM). Statistical significance was defined as *p* < 0.05. Data visualization was performed using GraphPad Prism software (version 9.5; GraphPad Software, San Diego, CA, USA).

## 3. Results

### 3.1. Growth Performance

**Figure 1:**
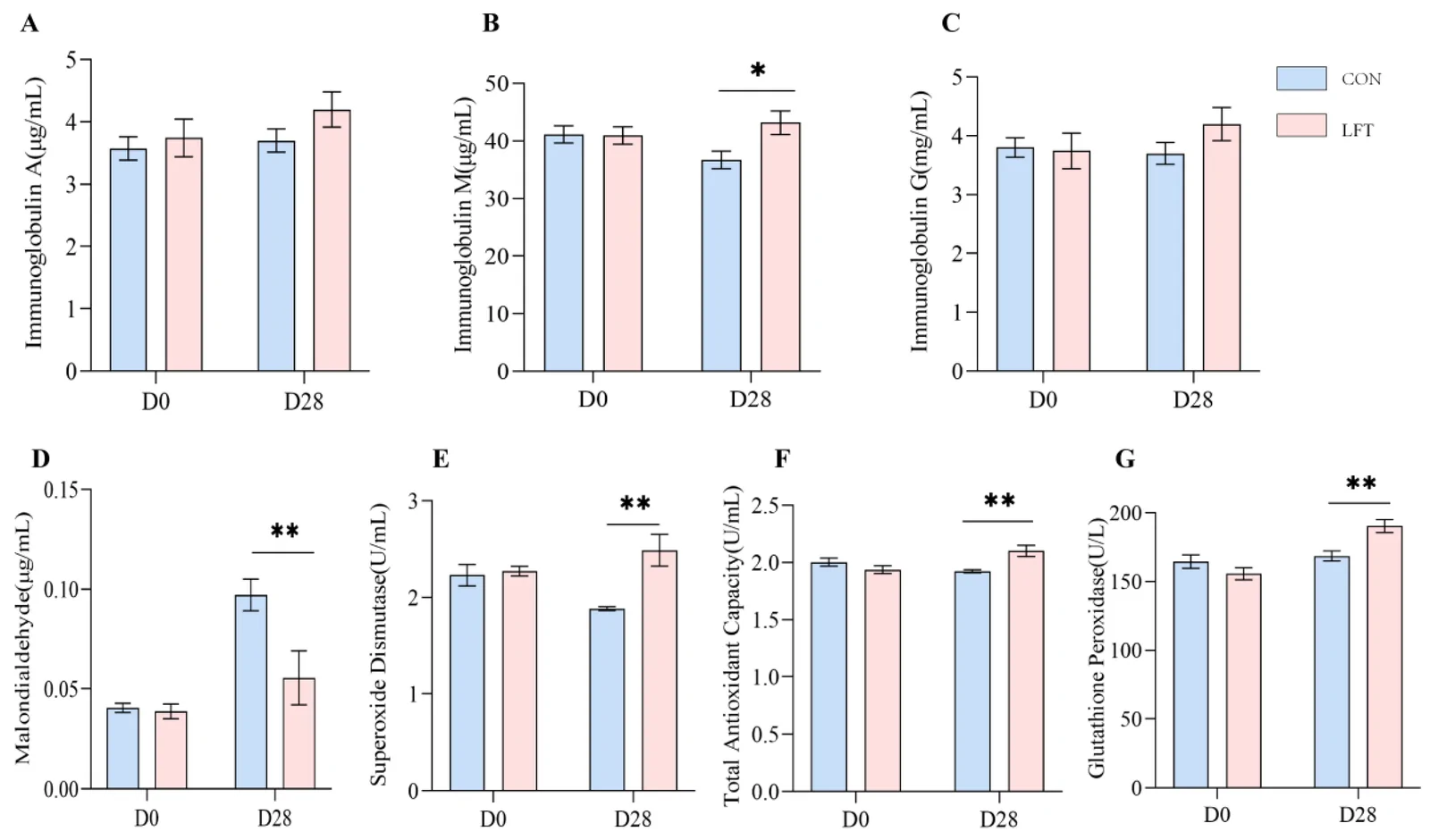
Effects of dietary supplementation with LF-OPN complex on serum immunoglobulins and antioxidant indices in kittens. (**A**) Immunoglobulin A (IgA); (**B**) immunoglobulin M (IgM); (**C**) immunoglobulin G (IgG); (**D**) malondialdehyde (MDA); (**E**) total antioxidant capacity (T-AOC); (**F**) glutathione peroxidase (GSH-Px); and (**G**) superoxide dismutase (SOD). Data are expressed as the mean ± SEM (*n* = 8). * *p* < 0.05; ** *p* < 0.01. CON, basal diet group; LFT, basal diet supplemented with LF-OPN.

The effects of dietary supplementation with LF-OPN complexes on kitten growth are presented in Table 2 and Figure 1. No significant differences in body weight (BW) or average daily gain (ADG) were observed between the CON and LFT groups during the entire experimental period (*p* > 0.05, Figure 1).

### 3.2. Serum Immunoglobulins and Antioxidant Parameters

The effects of dietary supplementation with LF-OPN complexes on serum immunoglobulins and antioxidant indices are shown in Figure 1. No between-group differences were detected at D0 for any measured variable (*p* > 0.05), confirming baseline comparability.

On D28, the serum IgM concentration was higher in the LFT group than in the CON group (*p* < 0.05, Figure 1B), whereas IgA and IgG concentrations did not differ (*p* > 0.05, Figure 1A,C). The MDA concentration was lower in the LFT group (*p* < 0.01, Figure 1D), whereas T-AOC and the activities of GSH-Px and SOD activity were higher (*p* < 0.01, Figure 1E–G).

### 3.3. Inflammatory Cytokines and Intestinal Barrier Function Parameters

**Figure 2:**
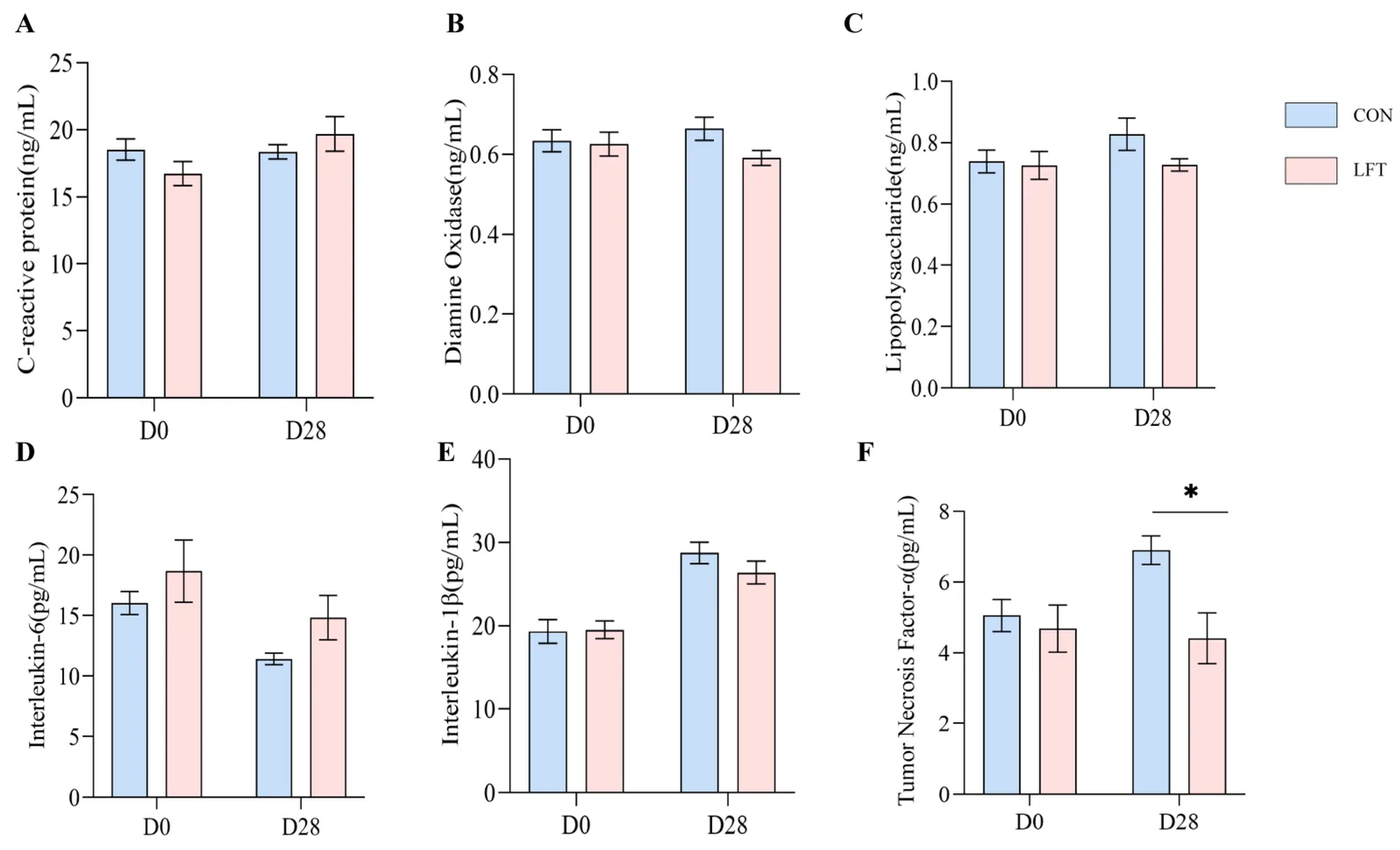
Effects of dietary supplementation with LF-OPN complexes on serum inflammatory and intestinal barrier-related indices in kittens. (**A**) C-reactive protein (CRP); (**B**) diamine oxidase (DAO); (**C**) lipopolysaccharide (LPS); (**D**) interleukin-6 (IL-6); (**E**) interleukin-1β (IL-1β); and (**F**) tumor necrosis factor-α (TNF-α). Data are expressed as the mean ± SEM (*n* = 8). * *p* < 0.05. CON, basal diet group; LFT, basal diet supplemented with LF-OPN.

On D28, the serum TNF-α concentration was lower in the LFT group than in the CON group (*p* < 0.05, Figure 2F). Serum IL-6 and IL-1β concentrations did not differ between groups (*p* > 0.05, Figure 2D,E). Serum LPS, DAO, and CRP concentrations also remained unchanged throughout the experimental period (*p* > 0.05, Figure 2A–C).

### 3.4. Fecal Microbiome Composition

**Figure 3:**
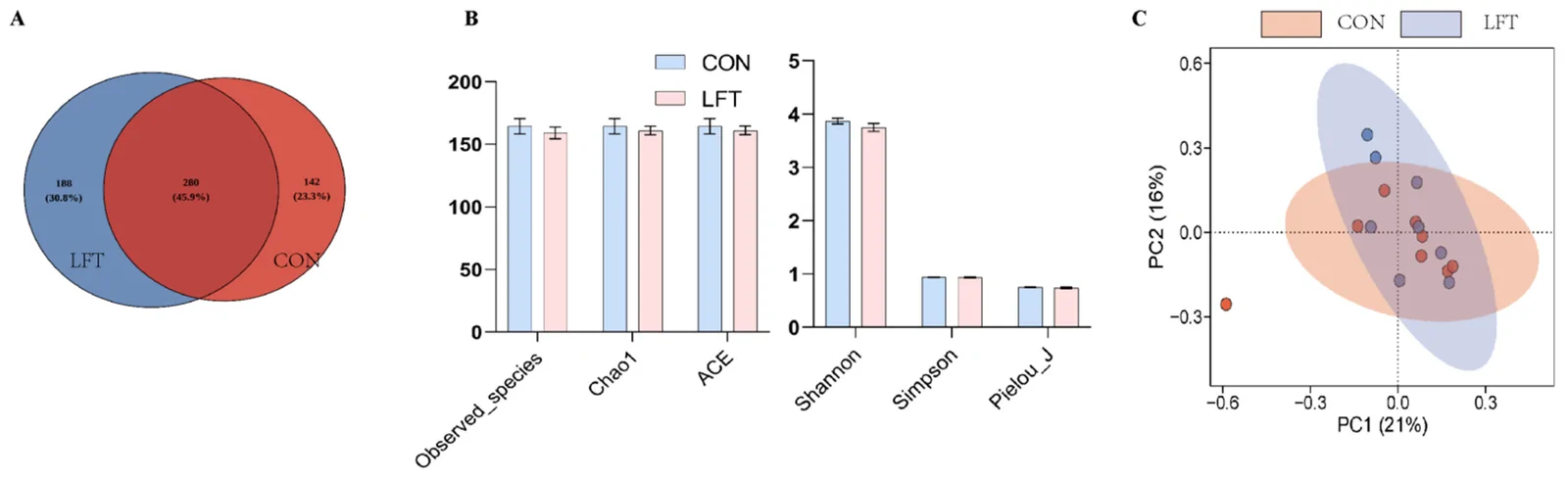
Effects of supplementation with LF-OPN complexes on the fecal microbiota composition and diversity in kittens. (**A**) ASV Venn diagram; (**B**) alpha diversity indices; and (**C**) principal coordinate analysis (PCoA) based on Bray–Curtis dissimilarity. Red indicates taxa enriched in the CON group, and green indicates taxa enriched in the LFT group. CON, basal diet group; LFT, basal diet supplemented with LF-OPN.

Fecal bacterial communities were characterized by 16S rRNA gene amplicon sequencing. The CON and LFT groups contained 142 and 188 unique amplicon sequence variants (ASVs), respectively (Figure 3A). Alpha diversity analysis, which reflects microbial richness and evenness, showed no significant differences in the Chao1, ACE, Shannon, or Simpson indices between groups (*p* > 0.05, Figure 3B). Principal coordinate analysis based on Bray–Curtis dissimilarity revealed a trend toward separation between the CON and LFT groups, with the first two principal coordinates explaining 21% and 16% of the variation, respectively (Figure 3C). However, PERMANOVA based on Bray–Curtis distances showed no significant difference in overall microbial community structure between the two groups (R^2^ = 0.0802, *p* = 0.187).

**Figure 4:**
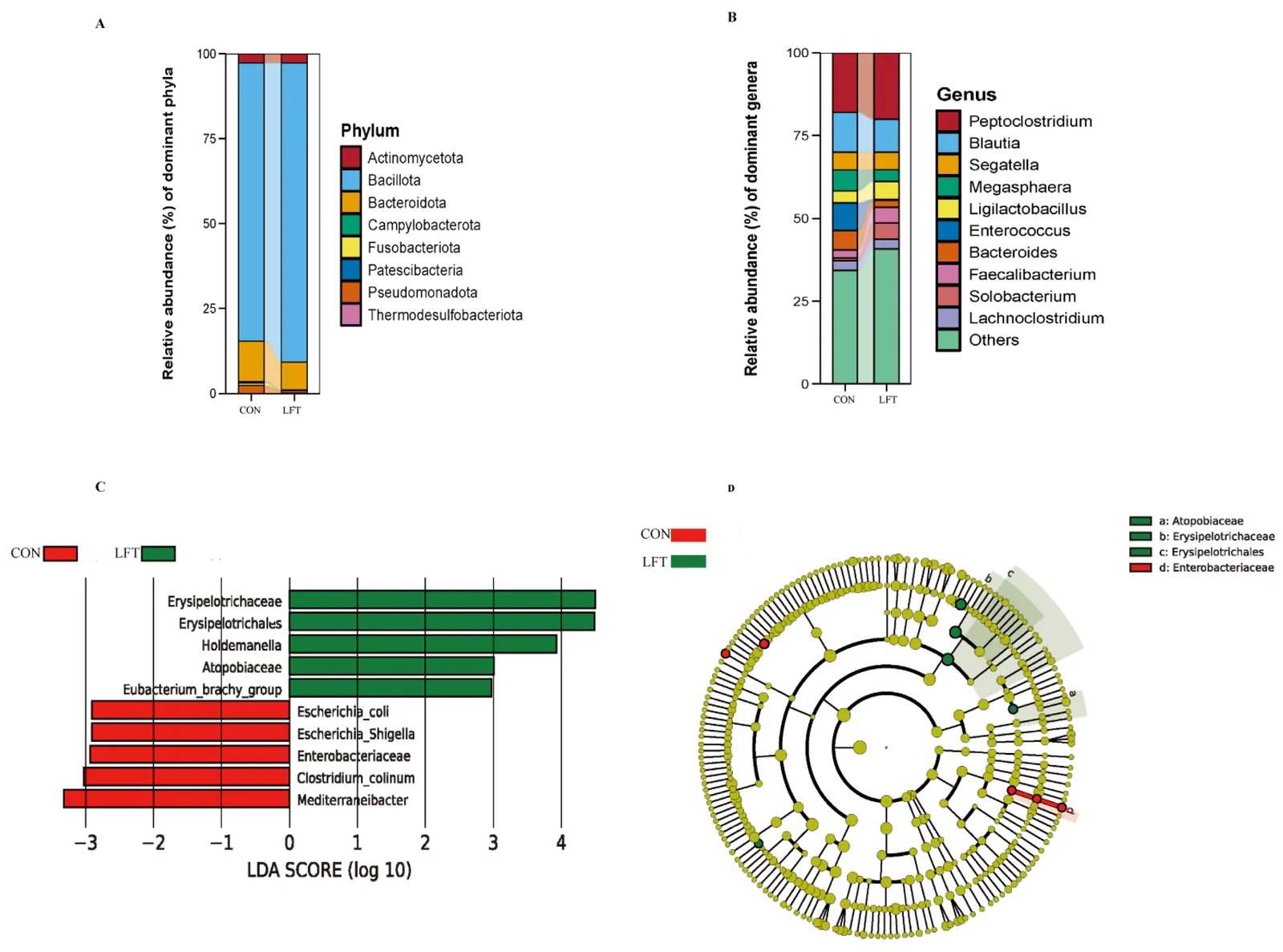
Effects of supplementation with LF-OPN complexes on the fecal microbiota composition and diversity in kittens. (**A**) Taxonomic composition at the phylum level; (**B**) relative taxonomic composition at the genus level; (**C**) LEfSe LDA score plot; and (**D**) taxonomic cladogram (LDA > 2; *p* < 0.05); Red indicates taxa enriched in the CON group, and green indicates taxa enriched in the LFT group. CON, basal diet group; LFT, basal diet supplemented with LF-OPN.

At the phylum level, both groups were dominated by Actinomycetota, Bacillota, Bacteroidota, Campylobacterota, Fusobacteriota, Patescibacteria, Pseudomonadota, and Thermodesulfobacteriota, with no significant differences in relative abundances between groups (*p* > 0.05, Figure 4A). At the genus level, the dominant genera included Peptoclostridium, Blautia, Segatella, Megasphaera, Ligilactobacillus, Enterococcus, Bacteroides, Faecalibacterium, Solobacterium, and Lachnoclostridium. The relative abundances of the 10 most abundant genera also did not differ significantly between the groups (*p* > 0.05, Figure 4B).

LEfSe analysis identified 10 differentially abundant taxa (Figure 4C,D). Taxa enriched in the LFT group included Erysipelotrichales (LDA = 4.48, *p* < 0.05), Atopobiaceae (LDA = 3.00, *p* < 0.05), Erysipelotrichaceae (LDA = 4.49, *p* < 0.05), Holdemanella (LDA = 3.92, *p* < 0.05), and the Eubacterium brachy group (LDA = 2.92, *p* < 0.05). Conversely, the CON group had higher abundances of Enterobacteriaceae (LDA = 2.93, *p* < 0.05), Mediterraneibacter (LDA = 3.32, *p* < 0.05), Escherichia-Shigella (LDA = 2.91, *p* < 0.05), Clostridium colinum (LDA = 3.02, *p* < 0.05), and Escherichia coli (LDA = 2.90, *p* < 0.05).

### 3.5. Correlation Between Fecal Microbes with Serum Parameters

**Figure 5:**
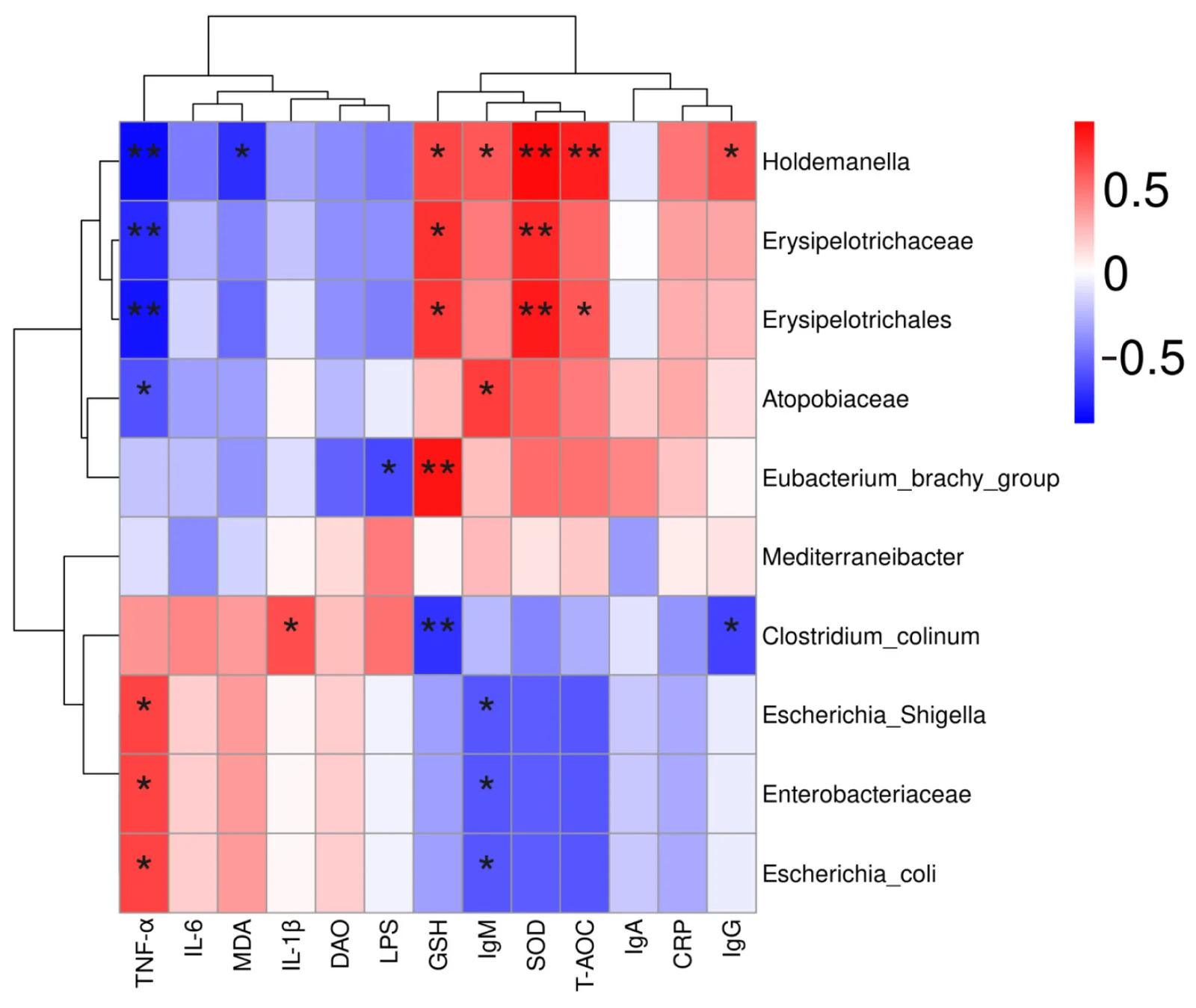
Correlation of fecal bacterial taxa with inflammatory and immune markers. The symbol (*) denotes statistically significant differences (* *p* < 0.05), the symbol (**) denotes statistically highly significant differences (** *p* < 0.01).

To further explore the relationships between bacterial taxa and serum parameters in kittens, Spearman’s correlation analysis was performed (Figure 5). Holdemanella was positively correlated with serum IgM, SOD, T-AOC, and IgA, and negatively correlated with MDA. Erysipelotrichaceae and Erysipelotrichales were positively correlated with serum IgM and T-AOC, and negatively correlated with TNF-α. Atopobiaceae was positively correlated with serum IgM and negatively correlated with MDA. Escherichia-Shigella, Enterobacteriaceae, and Escherichia coli were negatively correlated with serum IgM, SOD, and T-AOC, and positively correlated with MDA, TNF-α, and IL-6.

### 3.6. Fecal Short-Chain Fatty Acids

Fecal concentrations of acetate, propionate, isobutyrate, butyrate, isovalerate, valerate, and total SCFAs did not differ between the CON and LFT groups (*p* > 0.05, Table 3).

### 3.7. Flow Cytometric Analysis of Peripheral Blood Lymphocyte Subsets

**Figure 6:**
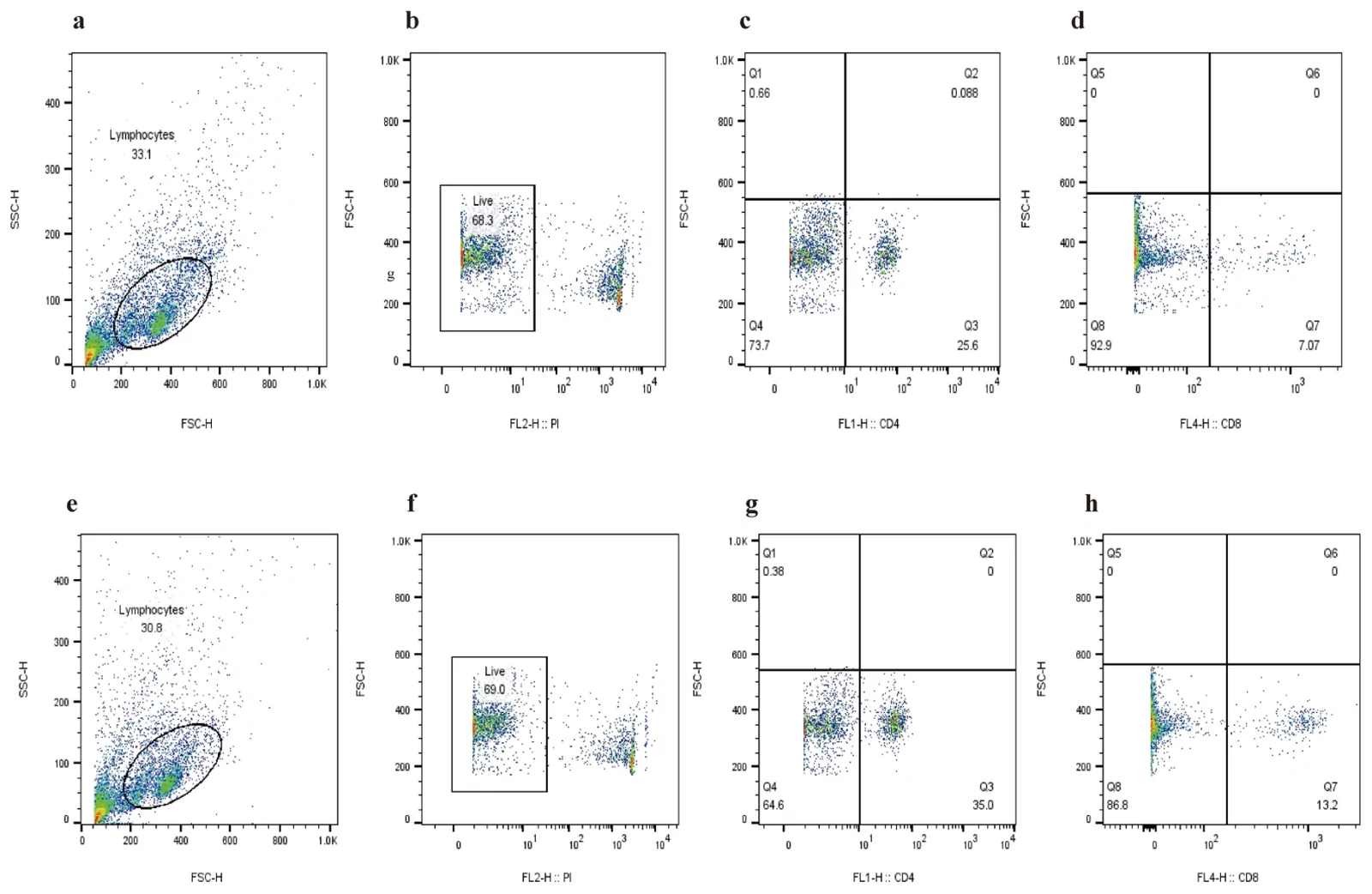
Effects of supplementation with LF-OPN complexes on peripheral blood lymphocyte subsets in kittens. The figure shows representative flow cytometry plots illustrating the gating strategy and subset quantification in both groups. Panels (**a**–**d**) show a representative sample from the CON group, and panels (**e**–**h**) show a representative sample from the LFT group. The gating strategy was as follows: (**a**,**e**) lymphocytes were first gated based on their forward scatter (FSC) and side scatter (SSC) properties. (**b**,**f**) Dead cells were excluded by gating on the Propidium Iodide (PI)-negative population (live cells). (**c**,**g**) CD4+ lymphocytes were determined within the Q2 quadrant of a CD4 vs FSC plot. (**d**,**h**) CD8 + lymphocytes were determined within the Q7 quadrant of a CD8 vs FSC plot. Values within each quadrant represent the percentage of cells within the parent live lymphocyte gate.

On D28, the proportion of CD4+ lymphocytes and the CD4+/CD8+ ratio were higher in the LFT group (*p* < 0.01, Table 4), whereas the proportion of CD8+ lymphocytes did not differ between groups (*p* > 0.05, Table 4). Representative gating plots are shown in Figure 6. Lymphocytes were first identified by FSC/SSC gating, and dead cells were excluded based on PI negativity; CD4+ lymphocytes were quantified in the Q2 quadrant of a CD4 vs FSC plot; and CD8+ lymphocytes were quantified in the Q7 quadrant of a CD8 vs FSC plot. All percentages are expressed relative to the live lymphocyte parent gate.

### 3.8. Transcriptomic Analysis of Peripheral Whole Blood

**Figure 7:**
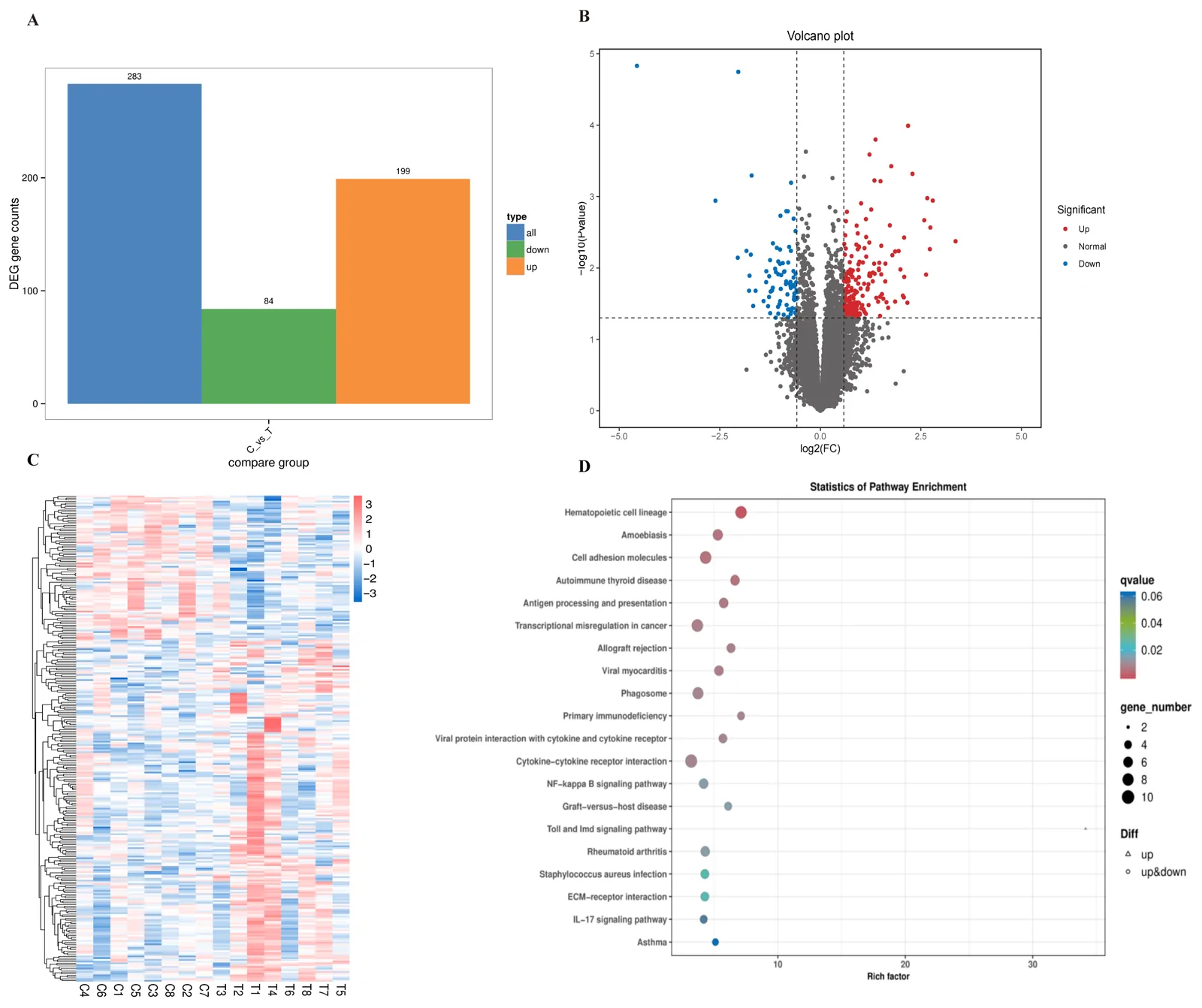
Effects of dietary supplementation with LF-OPN complexes on the peripheral blood transcriptome of British Shorthair kittens. (**A**) Number of differentially expressed genes (DEGs); (**B**) volcano plot; (**C**) hierarchical clustering heatmap; (**D**) bar chart of enriched KEGG pathway enrichment.

Whole-blood RNA sequencing was performed to assess transcriptional differences associated with LF-OPN supplementation. Using the screening criteria of *p* < 0.05 and a |log_2_FC| > 1.5, 283 differentially expressed genes (DEGs) were identified between the LFT and CON groups, including 199 upregulated and 84 downregulated genes in the LFT group (Figure 7A,B). Hierarchical clustering showed distinct expression patterns between the groups, with many DEGs associated with immune regulation (Figure 7C).

KEGG enrichment analysis identified 20 significantly enriched pathways (*p* < 0.05; Figure 7D), including hematopoietic cell lineage, antigen processing and presentation, cytokine–cytokine receptor interaction, NF-κB signaling, phagosome, IL-17 signaling, and cell adhesion molecules. Selected DEGs and their associated pathways are summarized in Table 5.

## 4. Discussion

Early life is a critical period for gastrointestinal and immune development, and young kittens may be particularly sensitive to nutritional and environmental challenges. In this study, a commercial LF-OPN preparation with an LF:OPN mass ratio of 8:1 was evaluated as a complete product. These observed effects are biologically plausible given the reported immunoregulatory and intestinal activities of LF and OPN, as well as the digestive stability and epithelial bioactivity of LF-OPN complex formats [22,24,30,31,32]. Nevertheless, because the study did not include LF-only and OPN-only groups or an uncomplexed LF + OPN group, all effects must be attributed to the tested preparation; neither the responsible component nor synergy can be determined.

The LFT group had a higher serum IgM concentration, whereas IgA and IgG concentrations were unchanged. Natural and early-response IgM contributes to pathogen recognition, clearance of altered self-components, and regulation of subsequent immune responses [33,34,35]. Dietary LF has been reported to increase serum IgM in weanling piglets [24,36], whereas OPN participates in type 1 immune responses, and OPN-enriched formula has been shown to alter cytokine and lymphocyte profiles in infants [37]. Thus, the increased serum IgM concentration in the LFT group may reflect contributions from both LF and OPN and suggests an association between the LF-OPN preparation and early humoral immune parameters in young kittens.

Body weight did not differ between the CON and LFT groups, suggesting that LF-OPN supplementation primarily affected other aspects of health rather than growth promotion. The lower serum TNF-α concentration in the LFT group is consistent with a randomized infant trial in which OPN-enriched formula reduced circulating TNF-α [29]. LF can interact with innate immune pathways, including Toll-like receptor and NF-κB signaling [38,39]. Notably, taxa enriched following LF-OPN supplementation, particularly Holdemanella, have independently been associated with reduced TNF-α concentrations and alleviation of intestinal inflammation [40]. Therefore, the most cautious interpretation is that the LF-OPN preparation was associated with a systemic immune profile characterized by greater early humoral responsiveness and lower basal TNF-α under the conditions of this study. Despite the significant reduction in serum TNF-α in the LFT group, IL-6 and IL-1β remained unchanged, suggesting that the observed inflammatory response pattern was selective rather than broad-spectrum.

LF-OPN supplementation increased serum SOD, GSH-Px, and T-AOC and decreased MDA, indicating improved antioxidant status. LF supplementation has been reported to increase serum SOD and T-AOC activities in piglets [41], enhance GSH-Px activity, and reduce serum MDA concentrations [42]. Maternal bovine LF supplementation has also been reported to increase SOD and GSH-Px levels in maternal tissues and circulation and to enhance antioxidant capacity in the brain tissue of newborn offspring [43]. Complex formation may preserve LF and OPN during digestion, but direct evidence that OPN or LF-OPN complexation specifically accounts for the antioxidant response remains limited. These findings indicate that LF-OPN supplementation elevated the antioxidant defenses and may help maintain redox homeostasis in young kittens; however, they do not identify the responsible component or mechanism. Serum LPS, DAO, and CRP showed no differences between the groups. At 3–4 months of age, the kittens’ intestinal barrier is largely established, and under non-pathological conditions, nutritional intervention alone may not induce marked changes in intestinal barrier function parameters.

The intestinal mucosal barrier and resident microbiota jointly contribute to intestinal and systemic homeostasis [2,12,44,45,46], and LF enhances host defense by modulating the microbial ecosystem and reinforcing the intestinal barrier [47]. In healthy kittens, dietary interventions may produce selective taxonomic shifts without restructuring the entire microbial community [2,48]. In the present study, alpha diversity did not differ between the CON and LFT groups, indicating that LF-OPN supplementation did not substantially alter fecal microbial richness or evenness. The relative abundances of the dominant phyla and genera were also unchanged, and PCoA showed similar overall community structures, although a trend toward separation in beta diversity was observed. LEfSe identified 10 differentially abundant taxa. The lower relative abundances of Enterobacteriaceae, Escherichia–Shigella, and E. coli in the LFT group may be relevant because Enterobacteriaceae can expand during inflammation and dysbiosis [49]. This pattern is also consistent with reports that LF can reverse antibiotic-induced dysbiosis by restoring beneficial bacteria and regulating microbial composition [50]. *Holdemanella* has been reported to alleviate intestinal inflammation, repair mucosal damage, and reduce TNF-α concentrations [40]. In addition to the antimicrobial properties of LF, orally administered OPN can bind bacterial LPS and attenuate TNF-α-driven inflammatory signaling; OPN may also promote SCFA-producing taxa that support intestinal barrier integrity [51,52]. Taxa enriched in the LFT group, including Holdemanella and Erysipelotrichaceae, have been associated with metabolic or inflammatory phenotypes in other hosts [53,54]. Taken together, the simultaneous reduction in serum TNF-α and fecal Enterobacteriaceae abundance may reflect a microbiota-associated response to LF-OPN supplementation.

Despite these taxonomic shifts, fecal SCFA concentrations were unchanged. Early-life LF supplementation has increased colonic SCFA concentrations in piglets [55], whereas studies in infants have reported variable effects of LF-containing formulas on fecal microbial metabolites [56,57]. Ex vivo fermentation models of LF-OPN complexes have also shown increased SCFA production [21]. Cats, as obligate carnivores, have a lower fermentative capacity than many omnivorous species, and both the amount and physicochemical properties of fermentable substrates strongly influence SCFA production [58,59]. In the present study, fermentable substrates were derived primarily from rice and potatoes; therefore, the low fermentable-fiber content of the diet and the relatively short intervention period may have limited detectable changes in fecal SCFAs. Future studies using diets supplemented with fermentable fibers, such as fructooligosaccharides or chicory inulin, would help determine whether LF-OPN-induced microbiota shifts translate into functional metabolic changes in kittens.

Flow cytometric analysis used a standardized gating strategy specific for feline cells. Following the methodology of Byrne et al. [60], live lymphocytes were identified based on forward scatter (FSC) and side scatter (SSC) properties and by excluding PI-positive cells, after which CD4+ and CD8+ populations were quantified. Although CD4 and CD8 can also be expressed by some feline monocytes and natural killer cells, FSC/SSC gating reduced contamination from the larger and more granular monocyte population. Within the live lymphocyte gate, LF-OPN supplementation increased the CD4+ lymphocyte proportion, whereas the CD8+ lymphocyte proportion remained unchanged. LF has been reported to modulate cellular immune indices in mice and dogs [25,27], and OPN-enriched formula has altered T-cell proportions during early life [28]. The CD4+/CD8+ ratio is commonly used as an indicator of immune maturation [1,61]. Its increase in the LFT group, together with the broad helper function of CD4+ T cells in antibody production and immune coordination [62], suggests that LF-OPN supplementation may support adaptive immune development. However, functional assays are required to confirm this interpretation.

Whole-blood transcriptomic analysis identified DEGs enriched in immune-related pathways, including hematopoietic cell lineage, phagosome, antigen processing and presentation, cell adhesion molecules, cytokine–cytokine receptor interaction, NF-κB signaling pathway, and IL-17 signaling. These pathways are related to the immune regulation [39,63,64]. Immunoregulatory effects of the LF-OPN complex have also been reported [21,22]. LF-OPN supplementation upregulated CXCL8, IL1R2, PTGS2, CEBPB, KIT, TNFRSF19, and CLEC7A, and downregulated CD8A, CD8B, and MMP1. The observed expression change in CXCL8 and CLEC7A are compatible with innate immune pathway previously linked to LF [65], but the present data do not establish direct regulation of these genes or enhance host defense by LF-OPN. Although the upregulation of PTGS2 and CEBPB may indicate myeloid activation, concurrent upregulation of the decoy receptor IL1R2 may limit IL-1 mediated inflammatory signaling [39]. However, the transcriptomic and phenotypic findings were not uniformly concordant. Whole-blood RNA profiles are influenced by both leukocyte composition and cell-intrinsic transcription, and transcript abundance does not necessarily parallel protein abundance or cellular function. For example, CD8A and CD8B transcript levels were lower despite an unchanged CD8+ cell proportion. OPN participates in immune regulation, including the modulation of hematopoietic cell lineage and cytokine–cytokine receptor interaction pathways [16,32,37]. Overall, these transcriptomic findings suggest potential associations with innate immune and immunoregulatory pathways; however, given the small sample size and the lack of independent RT-qPCR validation, they should be considered exploratory and hypothesis-generating, and the functional consequences of these transcriptional shifts remain to be determined.

LF-OPN preparation shows potential influence in changing oxidative status and immune-related indices; however, these findings should be interpreted cautiously. The results were limited to the tested LF-OPN preparation and further constrained by the small sample size of only eight kittens per group, the single-dose were tested and short-duration design, individual differences, and the experimental environment. Additionally, the microbiome correlation analyze and RNA-seq findings were exploratory, and transcriptomic results were not independently validated. These limitations reduce the strength of causal and mechanistic inferences and highlight the need for confirmation in larger, independently replicated studies. The specific contributions of each component need to be explored in subsequent studies. Nevertheless, future studies incorporating clinically relevant endpoints remain worthy of attention to provide more data for pet food development.

## 5. Conclusions

Dietary supplementation with a commercial LF-OPN preparation at 240 mg/kg body weight was well tolerated in kittens and did not adversely affect growth. Supplementation was associated with a higher serum IgM concentration, changes in antioxidant indices, lower serum TNF-α, a higher peripheral CD4+ proportion and CD4+/CD8+ ratio, selective changes in fecal bacterial taxa, and exploratory differences in immune-related whole-blood transcriptional pathways. These exploratory findings support further evaluation of the LF-OPN preparation as functional ingredients in early-life feline nutrition in well-controlled studies with larger cohorts and appropriate comparator groups.

These conclusions are limited by the small sample size, single-dose and short-duration design, and lack of LF-only, OPN-only and uncomplexed LF + OPN comparator group. Future studies incorporating larger cohorts, dose–response designs, direct characterization of complex stability, functional immune outcomes, and diets with controlled fermentable-fiber content will be required to determine whether the observed LF-OPN-associated effects are functionally significant and translate into meaningful clinical benefits, as well as to determine the optimal dosages for their application.

## Figures and Tables

**Table 1:** Composition and Nutrient Levels of the Basal Diet (air-dry basis).

Ingredient	%	Nutrient	%
Chicken meal	55.6	Dry matter	90.4
Fish meal	2.11	Crude protein	41.3
Rice	5.35	Crude fat	22.2
Poultry fat	9.73	Ash	8.67
Fish oil	2.13	Calcium	1.10
Potato starch	18.20	Phosphorus	0.80
Potato	4.85		
Taurine	0.24		
Chicken liver powder	0.70		
L-arginine	0.55		
Salt	0.49		
Mineral and vitamins premix ^1^	0.05		
Total	100		

^1^ Mineral complexes and vitamins per kilogram of milk powder: vitamin A (14,500 IU), vitamin D3 (1000 IU), vitamin E (156 IU), vitamin B1 (32.0 mg), vitamin B2 (30.0 mg), vitamin B3 (120 mg), vitamin B5 (88.0 mg), vitamin B6 (13.0 mg), vitamin B12 (0.20 mg), Fe (FeSO_4_) 100 mg, Cu (CuSO_4_) 7.00 mg, Co (CoSO_4_) 1.00 mg, I (CaI_2_) 20.0 mg, Mn (MnSO_4_) 20.0 mg, Zn (ZnSO_4_) 68.0 mg and Se (Na_2_SeO_3_) 0.50 mg.

**Table 2:** Effects of dietary supplementation with LF-OPN complexes on body weight in British Shorthair kittens.

Experimental Time (Day)	CON (BW, kg)	LFT (BW, kg)
D0	1.35 ± 0.10	1.31 ± 0.08
D7	1.44 ± 0.09	1.39 ± 0.09
D14	1.59 ± 0.11	1.46 ± 0.08
D21	1.74 ± 0.12	1.68 ± 0.11
D28	1.87 ± 0.12	1.82 ± 0.09

Data are expressed as the mean ± SEM (*n* = 8). CON = basal diet group; LFT = basal diet supplemented with LF-OPN group.

**Table 3:** Effects of dietary supplementation with LF-OPN complexes on short-chain fatty acid concentrations in British Shorthair kittens.

SCFA (μg/g)	CON	LFT
Acetate	2406.88 ± 151.27	2411.24 ± 84.88
Propionate	1930.04 ± 140.81	1929.22 ± 89.90
Isobutyrate	160.01 ± 13.07	144.55 ± 22.31
Butyrate	1147.50 ± 99.89	1024.08 ± 70.01
Isovalerate	291.89 ± 30.53	229.92 ± 35.67
Valerate	458.92 ± 62.38	398.25 ± 108.50
Total SCFAs	6407.00 ± 187.70	6266.63 ± 196.90

Data are expressed as the mean ± SEM (*n* = 8). Concentrations are expressed as µg/g wet feces. CON, basal diet group; LFT, basal diet supplemented with LF-OPN.

**Table 4:** Effects of dietary supplementation with LF-OPN complexes on lymphocyte subsets in British Shorthair kittens.

Lymphocyte Subset	CON	LFT
CD4+ (% of live lymphocytes)	21.76 ± 0.18	31.71 ± 0.21 **
CD8+/(% of live lymphocytes)	10.85 ± 0.23	11.21 ± 0.31
CD4+/CD8+ ratio	2.01 ± 0.04	2.84 ± 0.08 **

Data are expressed as the mean ± SEM (*n* = 8). ** *p* < 0.01. CON, basal diet group; LFT, basal diet supplemented with LF-OPN.

**Table 5:** Core differentially expressed genes (DEGs) and their enriched KEGG pathways in kittens following supplementation with LF-OPN complexes.

Gene	Regulation (LFT vs. CON)	KEGG Pathway	Biological Role/Significance
*CXCL8*	Upregulated	IL-17 signaling pathway	Neutrophil chemotaxis and activation
*IL1R2*	Upregulated	Cytokine–cytokine receptor interaction	Negative regulation of IL-1 signaling
*PTGS2*	Upregulated	NF-κB signaling pathway	Prostaglandin synthesis and inflammatory signaling
*CEBPB*	Upregulated	IL-17 signaling pathway	Transcriptional regulation of myeloid and inflammatory responses
*CD8A*	Downregulated	Antigen processing and presentation	T-cell co-receptor signaling and antigen recognition
*CD8B*	Downregulated	Antigen processing and presentation	CD8 co-receptor function in cytotoxic T-cell recognition
*KIT*	Upregulated	Hematopoietic cell lineage	Regulation of hematopoietic cell proliferation and differentiation
*TNFRSF19*	Upregulated	Cytokine–cytokine receptor interaction	TNF-receptor-family signaling
*CLEC7A*	Upregulated	C-type lectin receptor signaling	Pattern recognition in innate immunity
*MMP1*	Downregulated	IL-17 signaling pathway	Extracellular-matrix remodeling

CON, basal diet group; LFT, basal diet supplemented with LF-OPN (*n* = 8 per group).

## Data Availability

The original contributions presented in this study are included in the article. Further inquiries can be directed to the corresponding author.
